# Dietary Phenethyl Isothiocyanate Protects Mice from Colitis Associated Colon Cancer

**DOI:** 10.3390/ijms18091908

**Published:** 2017-09-06

**Authors:** Yi Liu, Moul Dey

**Affiliations:** Department of Health and Nutritional Sciences, Box 2275A, South Dakota State University, Brookings, SD 56007, USA; liu.yi1@mayo.edu

**Keywords:** phenethyl isothiocyanate (PEITC), colitis associated colon cancer

## Abstract

We have previously reported alleviation of dextran sodium sulfate (DSS)-induced ulcerative colitis signs in phenethyl isothiocyanate (PEITC)-treated mice. Here we investigated chemoprotective activities of PEITC in mice with Azoxymethane-DSS induced colitis associated colon carcinogenesis. We also examined the molecular mediators associated with the PEITC effects using relevant cell lines. A 0.12% PEITC-enriched mouse-diet reduced mucosal and submucosal inflammation as well as glandular atypia by 12% and the frequency of adenocarcinoma by 17% with a concomitant improvement in overall disease activity indices compared to the diseased control group. Lipopolysaccharide-induced in vitro up-regulation of key mediators of inflammation, immune response, apoptosis, and cell proliferation were attenuated by 10 μM PEITC. Three of these mediators showed concentration-dependent reduction in respective mRNAs. Furthermore, PEITC inhibited Nuclear factor kappa B1 (NFκB1) proteins in a concentration-dependent manner. The *NFκB1* mRNA expression inversely correlated (*r* = −0.940, *p* = 0.013) with tri-methylation of lysine 27 on histone 3 near its promoter region in a time-dependent manner. These results indicate that PEITC may slow down the development of colon carcinogenesis in an inflammatory intestinal setting which is potentially associated with epigenetic modulation of *NFκB1* signaling.

## 1. Introduction

Colorectal cancer (CRC) is second among various cancers in terms of causing deaths in the United States, and it resulted in an estimated healthcare expenditure of $14 billion in 2014. Over 90% of localized CRC, when diagnosed early, would have a 5-year survival rate. However, early detection is less common due to lack of compliance to screening regimens. For late diagnosed metastatic disease, survival rate drops to 8–12%. Cytotoxic chemotherapy is the only available treatment option for unresectable metastatic disease, to which the initial response frequently tapers off due to ensuing resistance within six months [1]. Due to this and other reasons, among cancer patients, the use of alternative treatments ranges between 30% and 75% worldwide and frequently includes dietary approaches [2].

The risk of developing CRC increases in patients with inflammatory bowel disease (IBD) [3]. IBD is a chronic inflammatory disorder, characterized by cytokine imbalance and transcription signaling pathways activation [4]. An estimated 3 million individuals in the United States suffered from IBD in 2015 and the numbers affected are rising (Available Online: https://www.cdc.gov/ibd/data-statistics.htm). Currently, there is no medical cure for IBD and therefore, patients require a lifetime of care.

Phenethyl isothiocyanate (PEITC) is—derived from common cruciferous vegetables. Our team and other laboratories have reported earlier that PEITC has anti-inflammatory and chemopreventive effects against various cancers [2,5,6,7,8,9,10]. PEITC has also been tested in clinical trial (NCT00691132). However, in the previous studies, PEITC has been mostly used as an oral-use natural product and not as a dietary ingredient. Furthermore, the efficacy of PEITC as a chemoprotective agent for CRC is comparatively less established compared to its chemopreventive and/or chemoprotective effects against other types of cancers [11]. One study showed PEITC offered protection against development of colonic aberrant crypt foci in rats [12], which was later contradicted by another study [13]. A mice study showed PEITC’s anticarcinogenic effect may be mediated through increased apoptosis in the colon but did not correlate such effects with its anti-inflammatory properties [14]. Here we investigated if a diet enriched with PEITC may help protect against CRC in an inflammatory bowel setting. We used a combination of azoxymethane (AOM) and dextran sodium sulfate (DSS) induced colitis associated colon carcinogenesis (CAC) model [15] as an extension of our previous work with PEITC in a DSS induced IBD model. In the previous work, we have shown that oral administration of PEITC was effective at remitting acute and chronic signs of ulcerative colitis (UC) in mice [16].

## 2. Results

### 2.1. Phenethyl Isothiocyanate (PEITC) Treatment Improved Colon Cancer Associated Clinical Signs in Mice

**Figure 1:**
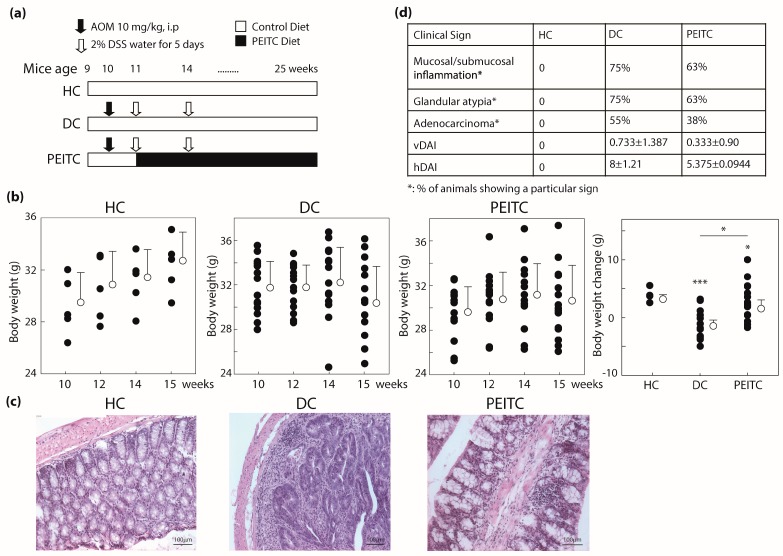
Phenethyl isothiocyanate or PEITC protects experimental mice from AOM/DSS induced colitis associated colon cancer. (**a**) Experimental design; (**b**) Body weights; (**c**) Representative H&E stained colonic sections (×100) from each group; (**d**) Table of clinical sign (**a**) In vivo experimental design; (**b**) Body weight changes shown from week 10 to 15 (all panels); (**c**) Representative H&E staining from each experimental mice group showing presence of goblet cells (HC, PEITC groups), absence (HC) or minimal presence (PEITC) of inflammatory cell aggregates, and presence of precancerous and cancerous lesions (DC), scale bars measure 100 μm; (**d**) Tabular summary of clinical signs. *n* = 15 (except 5 for HC), * *p* < 0.05, *** *p* < 0.001. PEITC, phenethyl isothiocyanate; HC, healthy control; DC, disease control; AOM/DSS, azoxymethane/dextran sodium sulfate.

To investigate the role of PEITC in inflammation-induced colon carcinogenesis, we randomly divided mice into three groups including two control groups, healthy control (HC) and disease control (DC), and one PEITC-diet test group (Figure 1a). Body weight (B.W.) loss is a common hallmark of DSS-induced UC as well as CAC [17]. As expected, steady body weight increase was hindered due to disease induction (DC, PEITC-diet, *p* = 0.00015 and 0.026 respectively) compared to mice that remained healthy throughout the experiment. The slowdown in weight gain was, however, partially recovered when mice received 0.12% PEITC enrichment in diet (*p* = 0.043, Figure 1b). It is noteworthy to mention that this partial recovery was achieved even when average food intake per mouse per day in the PEITC group (5.2 g/30 g B.W.) was slightly lower than in the other two groups (5.8 g/30 g B.W.), which could be due to difference in palatability, potentially arising from PEITC-enrichment. Food intake of all mice during the experiment was close to the reported average intake of 27 mice strains of 5.7 g/30 g B.W. per day [18]. PEITC concentration in diet was arbitrarily determined for this proof of concept study. Only one other study reported addition of PEITC in diet for ad libitum consumption in experimental mice at 0.05%, but it did not establish a reason why that specific concentration was used [14]. We have previously reported in vivo use of 75 mg/kg of 97% pure PEITC administered by oral gavage for effective amelioration of DSS-induced acute and chronic colitis [16]. However, in the present study, we used a higher dosage taking into consideration that chemotherapies for cancer treatment are typically used in high concentrations. Also, since PEITC activity may be lost due to heat during cooking [19], precautions were taken to minimize heating during addition of PEITC to the chow by LabDiet (St. Louis, MO, USA). No obvious signs of toxicity, including excessive weight loss, were observed in the experimental mice.

Histopathological assessment of colon sections from experimental mice revealed a number of cellular changes but no bulk tumor incidence after 15 weeks of single 10 mg/kg AOM injection. There is one existing report where tumor incidence within a similar experimental setup was reported after 20 weeks of an unspecified dose of AOM injection in the same mice strain [14]. In the colonic sections of DC group, extensive infiltration of submucosa and superficial muscularis by a mixed population of inflammatory cells (lymphocytes and macrophages) were observed as well as glandular atypia and signs of adenocarcinoma (representative micrographs shown in Figure 1c). Loss of goblet cells and crypt structure were widespread in the DC group. Rectal parts of the colons were generally characterized with the worst pathology. In the bowels of PEITC-diet group, crypt structures with intact goblet cells were frequently visible with less frequent and less severe signs of inflammation compared to the DC group. In the PEITC-diet group, presence of hyperplastic squamous epithelium and increased mitotic index was sporadic with fewer adenocarcinoma (Figure 1c,d). These differences in histopathological features when scored in a blinded manner resulted in a 33% (*p* = 0.11) lower histological Disease Activity Index (hDAI) in the PEITC-diet group comparing with the DC group. The criteria included for scoring visible Disease Activity Index (vDAI) included rear end inflammation, rectal bleeding, and stool consistency and was 54% lower in the PEITC-diet group compared to DC mice (Figure 1d). Taken together, PEITC-diet attenuated inflammation and colon carcinogenesis in experimental mice.

### 2.2. Concentration Dependent Inhibition of Cell Survival by PEITC

In the animal study, PEITC-diet group showed decreased inflammation in the colon epithelium. Subsequently, we investigated whether PEITC had an inhibitory effect on cell proliferation using two relevant cell types, inflammatory macrophage cells (RAW264.7) and colon tumor derived epithelial cells (SW480). The concentration range of PEITC was selected based on published studies [5,16]. At 6 h, more than 90% of RAW264.7 and SW480 cells showed high cell viability for all concentrations suggesting PEITC was non-cytotoxic to cells with short time PEITC exposure (Table 1). However, at 24 h, significant concentration-dependent loss in cancer cell viability was observed (Table 1). For all downstream investigation into key mediators of PEITC activity, we used the non-cytotoxic window of exposure of 5 to 8 h at or below 15 μM PEITC. It is possible that longer PEITC exposure induced apoptosis in both cell types resulted in the concentration-dependent inhibition of cell survival. These observations are in line with our previous work in which we have shown that PEITC is associated with higher expression of pro-apoptotic genes in human colon epithelial cells [5]. Also, there is at least one report where PEITC administration increased in vivo expression of pro-apoptotic proteins in mice colons [14].

### 2.3. Reduced Expression of Inflammatory Mediators after PEITC Exposure

**Figure 2:**
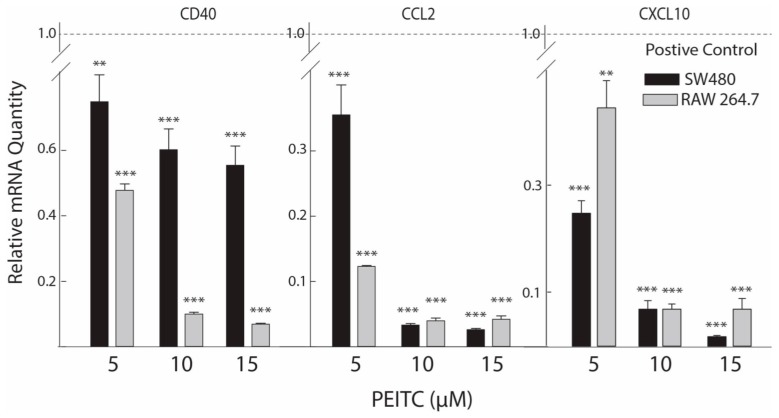
PEITC concentration-dependent mRNA expression of chemokines/cytokines. The effects of PEITC treatments were measured by the relative mRNA quantity expressed by chemokine/cytokine genes in the treated cells. Lower values represent greater inhibitory effects. Values are mean ± S.E. (*n* = 3). ** *p* < 0.01, *** *p* < 0.001 compared with positive-control cells (LPS activation normalized to a value of 1.00). LPS, lipopolysaccharide.

**Figure 3:**
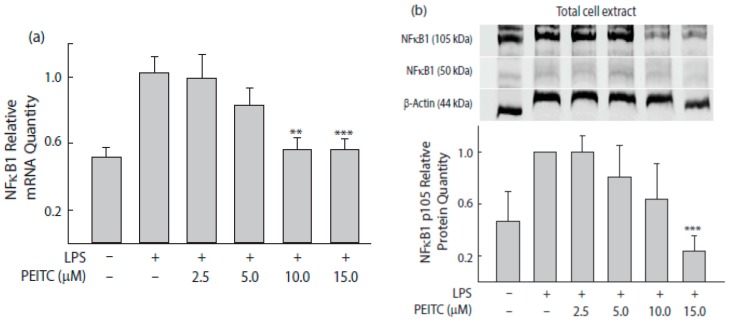

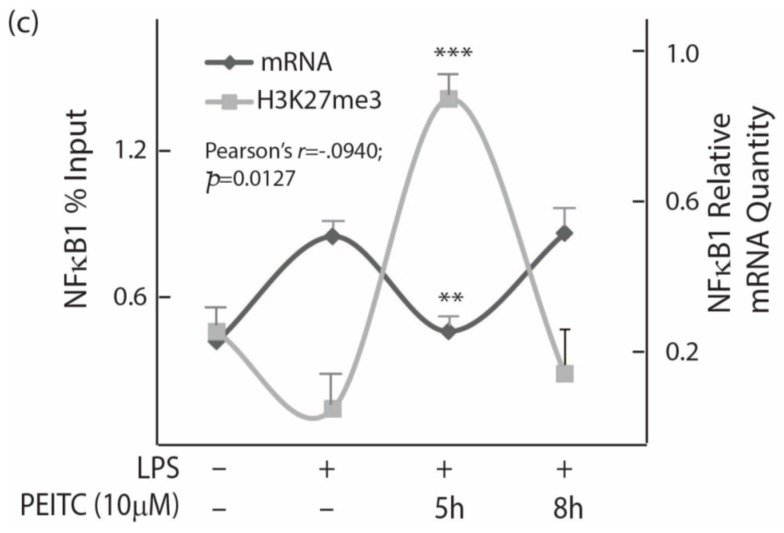
PEITC concentration- and time-dependent *NFκB1* suppression in colon epithelial cells. (**a**) The relative *NFκB1* mRNA quantity in the treated cells. Cells were treated with PEITC for 5 h before adding LPS for an additional 4 h incubation. Negative control cells did not receive any treatment, while positive control cells received LPS stimulation only. *GAPDH* was used as an internal control for mRNA expression. Values are mean ± SEM (*n* = 6); (**b**) Immunoblot analyses showing suppression NFκB1 in response to PEITC treatments. Expression levels were normalized to β-actin. Values are expressed as means ± SEM (*n* = 3) of three separate experiments; (**c**) Effect of PEITC (10 μM) treatment on *NFκB1* mRNA levels and on H3K27me3 methylation state in a time-dependent manner. Histone H3 methylation changes on *NFκB1* promoter were measured by chromatin immunoprecipitation using an anti-H3K27me3 antibody and followed by qPCR. Data points represent the average % input ± SEM (*n* = 3) from each experiment. ** *p* < 0.01, *** *p* < 0.001 compared with positive control.

In IBD patients, chronic inflammation may help augment the development of CRC. It is suggested that various cytokines released by epithelial and immune cells play a critical role in the pathogenesis of CAC [20]. We carried out RT-qPCR analyses on selected inflammatory mediators that are either directly part of or interact closely with the Nuclear Factor kappaB *(NFκB)* signaling network (Table 2). PEITC attenuated expression of all the selected genes, four of which were further down regulated in a concentration dependent manner (Figure 2 and Figure 3a), indicating anti-inflammatory effects of non-cytotoxic concentrations of PEITC in cell types relevant to intestinal inflammation in mice. At 10 μM concentration, PEITC significantly attenuated cytokine *CD40* (50.8% and 91.8%, *p* < 0.001), chemokine (C–C motif) ligand 2 (*CCL2*, 97.6% and 97.2%, *p* < 0.001), and chemokine (C–X–C motif) ligand 10 (*CXCL10*, 93.0% and 93.0%, *p* < 0.001) as well as transcription factor *NFκB1* (33.3% and 81.7%, *p* < 0.001) mRNAs compared with positive control cells (lipopolysaccharide induced and vehicle treated) in colon epithelial and macrophage cells, respectively (Figure 2 and Figure 3a). The data on *CXCL10* presented here reaffirms our previous report where we only showed its response to PEITC in macrophage cells [16]. For *CD40*, the response to PEITC was higher in the macrophage cells, while *CXCL10* and *CCL2* responded in a similar manner in both cell types.

### 2.4. PEITC Exposure Attenuates NFκB1 Transcription Factor That Inversely Correlate with Tri-Methylation Levels of Lysine 27 on Histone 3 near Its Promoter

Chronic inflammation linked with cancer is associated with NFκB and its effectors’ pathways. The concentration-dependent suppression of *NFκB1* mRNA upon PEITC exposure was further substantiated by concentration dependent NFκB1 protein inhibition (Figure 3b). Since tri-methylation of H3 at lysine 27 (H3K27me3) is frequently associated with transcriptional repression of genes, we further evaluated changes in H3K27me3 levels near the *NFκB1* promoter region and observed an inverse association (*r* = −0.940, *p* = 0.013) with a time-dependent changes in *NFκB1* mRNA expression (Figure 3c). At 5 h PEITC exposure time, the expression level of *NFκB1* mRNA was the lowest with corresponding highest level of H3K27me3 detected near its promoter. A PEITC-concentration dependent increase of H3K27me3 was also observed.

## 3. Discussion

About 75% of the currently available anticancer drugs have natural product origins. A strong inverse relationship between the incidence of cancer and dietary intake of cruciferous vegetables was reported by several epidemiological studies. Gluconasturtiin present in many cruciferous vegetables is converted to PEITC by the action of the enzyme myrosinase while chewing [11]. It is also reported that gut microbiota is capable of producing isothiocyanates from cruciferous vegetables in the colon [21]. We have previously reported in vivo effects of PEITC as an anti-inflammatory and cancer stem-like cell inhibiting agent [2,16,22]. In these studies, PEITC was orally administered more like a drug. In the current study, we administered PEITC as an ingredient in the diet, much like a functional food agent, and evaluated its chemoprotective potential within an inflammatory bowel setting. This is the first study where dietary PEITC-induced chemoprotection is evaluated in a CAC mouse model followed by investigation of its mechanistic underpinnings related to a potential epigenetically coordinated anti-inflammatory effect. It is generally accepted that inflammation-related molecules and pathways are useful targets for the prevention and treatment of cancer [23,24,25]. Furthermore, dietary factors can modulate in vivo epigenetic signatures and, therefore, susceptibility to diseases [5,26]. Of particular interest, in the context of inflammatory gene expression modulation by dietary agents is the tri-methylation of H3 at lysine 27 (H3K27me3), which is frequently associated with transcriptional repression and gene silencing [27,28].

Several mechanisms have been proposed to explain the anticancer effects of PEITC including its ability to induce cell cycle arrest and apoptosis. Another important mechanism proposed was PEITC-mediated generation of reactive oxygen species that leads to its cytotoxic effects, especially in cancer cells. However, less has been reported on PEITC interaction with *NFκB1* signaling in the specific context of CAC.

Chronic inflammation has been implicated as a contributor of CRC, but the exact underlying mechanism(s) has remained elusive. However, growing evidence points to the NFκB-signaling pathway that regulates cell proliferation, apoptosis, and inflammatory responses. NFκB activation was reported in 40% of colon tumors and in the majority of CRC cell lines. The most commonly studied *NFκB* complex is the RelA:p50 heterodimer [29,30,31]. However, one study has specifically shown that the variant NFκB1 (NFκB p105 subunit) is also associated with CRC risk in humans [32,33]. Furthermore, a protein-protein interaction network study reported NFκB1 as one of the seven observed key proteins associated with IBD signaling network [34]. Since this study is based on inflammation-induced colorectal cancer, which typically arises from inflammation and the development of adenoma to adenocarcinoma, we attempted to evaluate effects of PEITC on *NFκB1* transcription factor and three related signaling mediators and target genes. One of these is *CD40*, which was reported to be expressed in 110 human colon cancer tissue samples but not in normal colon tissues and is known to be associated with apoptosis and immune response [35]. The *CCL2* is a proinflammatory chemokine reported to regulate the recruitment of myeloid cells into inflamed sites and tumors that promotes cancer development and progression [36]. Finally, clinical data demonstrates that expression of the *CXCL10* is associated with increased metastatic potential in colon cancer, as well as in other types of cancer patients [37]. At non-cytotoxic concentrations, PEITC significantly suppressed mRNA expression of all four pro-inflammatory mediators.

In summary, using an AOM-initiated and DSS-promoted CAC model, we show that a PEITC-enriched diet may slow down colon cancer development in experimental mice. Follow-up molecular investigations in CAC-relevant cells showed inhibition of cancer cell proliferation by PEITC at higher concentrations. At non-cytotoxic concentrations, a key pro-inflammatory transcription factor *NFκB1* and associated expression of interacting genes were down regulated after PEITC exposure. The PEITC associated reduced expression of the *NFκB1*, correlated with changes in a gene-suppressing histone modification mark. This indicates at least partially that PEITC activity leads to a potential epigenetic regulation of *NFκB1* that relates to its anti-inflammatory effects. However, since the epigenetic repression appeared to be reversible with time, further investigation is warranted in the future to understand if there are parallel inflammation-regulatory mechanisms induced by PEITC. Together, the results from this otherwise small scale pilot study provide a basis for further investigation of PEITC as a chemoprotective agent for CAC.

## 4. Materials and Methods

### 4.1. Materials

For cell culture, Dulbecco’s Modified Eagle’s Medium (DMEM) was purchased from HyClone (Logan, UT, USA), fetal bovine serum (FBS), and TrypLE were purchased from Invitrogen Gibco (Grand Island, NY, USA). Human interferon-γ (IFNγ) was purchased from R&D Systems (McKinley, MN, USA). Dimethyl sulphoxide (DMSO), lipo-polysaccharide (LPS, from *Escherichia coli*, O55:B5), penicillin/streptomycin, and phenethyl isothiocyanate (PEITC) were purchased from Sigma-Aldrich (St. Louis, MO, USA). The β-actin antibody was purchased from Santa Cruz Biotechnology (Santa Cruz, CA, USA), the NFκB1antibody was from Millipore (Billerica, MA, USA), and the Dylight 800 anti-rabbit secondary antibody came from Li-Cor Biosciences (Lincoln, NE, USA). Also, for chromatin immuno-precipitation (ChIP) assay, the enzyme micrococcal nuclease (MNase), as well as anti-trimethyl-Histone H3 Lys27 and rabbit IgG for negative control samples were supplied by Cell Signaling (Beverly, MA, USA), Upstate Biotechnology (Billerica, MA, USA), respectively. IDT DNA Inc., (Coralville, IA, USA) synthesized all oligonucleotides. Dextran Sulfate Sodium (36,000–50,000 Da) was obtained from MP Biomedicals (Solon, OH, USA).

### 4.2. In Vivo Animal Studies

Animal study was approved by Institutional Animal Care and Use Committee of South Dakota State University (Approval#10-007A, approval date 1 January 2010) for the protocol titled: An alternate therapy for treatment of IBD and prevention of colon cancer) and followed the guidelines set forth by the Animal Welfare Act and the National Institutes of Health Guide for the Care and Use of Laboratory Animals. Eight weeks old C57BL/6J male mice (Charles River, Wilmington, MA, USA) were used. The housing for the mice maintained a constant temperature of 24–26 °C with 12 h light/dark cycle with free access to food and drinking water. Carbon dioxide inhalation was used for euthanasia after 16 weeks treatment. After terminal collection of the colons, they were flushed with sterile PBS, measured, and subsequently stored in 10% neutral buffered formalin for further processing. Hematoxylin and Eosin (H&E) stained cross-sections (~6 μm) were subjected to histological evaluation (×100 magnification) in a blinded manner.

The mice were randomized into three groups, which are described in Figure 1a. For HC and DC groups, mice were given regular diet (5015 diet, LabDiet, St. Louis, MO, USA) for 16 weeks. The same diet was supplemented with 0.12% PEITC for the PEITC-diet test group which they started after first two weeks. PEITC was added to the diet at the facility where the control diet was manufactured using methods that would prevent heat-denaturation of PEITC (LabDiet, St. Louis, MO, USA). DC and PEITC-diet groups were given AOM intra-peritoneally at the beginning of second week. Additional inflammation was induced twice by freshly prepared 2% DSS (ad libitum) in drinking water for five days at the beginning of third week and sixth week, respectively following previously published protocols [38,39]. Mice were observed and recorded individually for their general health conditions and clinical sign assessment, including body weight, vDAI and hDAI as described in Dey et al. [22]. All mice were weighed before and after intraperitoneal injection with AOM and DSS cycles.

### 4.3. Cell Culture and PEITC Treatments

The mouse monocyte/macrophage cell line, RAW 264.7 (ATCC TIB-71), and human colon cancer cell line, SW480 (ATCC CCL-228), were purchased from ATCC (ATCC, Manassas, VA, USA) and cultured and treated as previously described by Dey et al. [16] and Liu et al. [5], respectively. Cells were grown in DMEM supplemented with 10% FBS, 1% penicillin (25 U/mL)/streptomycin (25 μg/mL) in a 95% air/5% CO_2_-humidified atmosphere at 37 °C. Briefly, RAW264.7 cells were treated with PEITC or DMSO (as a negative control) at a pre-determined dose for 6 h before elicitation with 1 μg/mL of LPS without IFNγ priming. SW480 cells were pretreated with IFNγ 10 ng/mL or control medium (as a negative control) for 12 h, and treated with PEITC or DMSO for 5 h and then stimulated with LPS 10 ng/mL for 4 h. PEITC treatments were performed at 5, 10 and 15 μM concentration. Relative number of viable cells were measured using Cell Proliferation Assay kit (MTS, 3-(4,5-dimethylthiazol-2-yl)-5-(3-carboxymethoxyphenyl)-2-(4-sulfophenyl)-2*H*-tetrazolium, inner salt; Promega, Madison, WI, USA) following the manufacturer’s instructions.

### 4.4. Total RNA Extraction, Purification, and cDNA Synthesis and Real-Time Quantitative PCR

RNA extraction, purification, cDNA synthesis and real-time PCR were performed as previously described by our group [5,26]. All samples were run in duplicate. Gene-specific primers used in the current study are described in Table 3. Calculations of relative gene expression levels were performed using the 2^−ΔΔ*C*t^ method [40]. The mRNA data presented in Table 2 and Figure 2 were taken from separate experiments.

### 4.5. Western Blot Analysis

For immunoblot analyses, IFNγ-primed PEITC-treated SW480 cells were activated with LPS for 4 h and harvested using RIPA lysis buffer. The protein concentrations were determined using Pierce BCA Protein Assay kit (Thermo Scientific, Rockford, IL, USA). Proteins (35–50 μg/lane) were separated by 12% SDS-PAGE prior to electrotransfer on polyvinyldene difluoride (PVDF) membranes (Thermo Scientific, Rockford, IL, USA). Blocking was acheived with 5% skim milk for 1 h. Incubation with primary antibodies occurred at 4 °C overnight, after which incubation with Dylight 800 anti-rabbit secondary antibody occurred for 1 h, followed by washing 3 times in PBS/T (0.1% Tween20 in PBS) at room temperature. Blots were imaged after rinsing in PBS containing 0.1% Tween20 followed by quantitative analyses using an Odyssey infrared imaging system (Li-Cor).

### 4.6. ChIP Assay in Cell Culture

ChIP assay was performed as described by Liu et al. [5]. Briefly, cells were lysed and treated with MNase (Cell Signaling, Beverly, MA, USA) to obtain DNA fragments of 300–800 bp that were then immunoprecipitated with target antibodies (anti-trimethyl-Histone H3 Lys27 and rabbit IgG) at 4 °C overnight. Extraction of immunoprecipitated DNA fragments using protein-A sepharose (Sigma-Aldrich) followed next prior to purification using DNA purifying slurry (Diagenode, Denville, NJ, USA). About 200 ng of purified DNA template was then amplified real-time PCR. Promoter-specific primers were used and are listed below (Table 3). Data was expressed as a percentage of the input DNA.

### 4.7. Statistical Analysis

Data are expressed as mean ± SEM. We performed one-way analysis of variance (ANOVA) to determine the significance between groups followed by the post-hoc Dunnett test. Intergroup comparisons in western blot and ChIP assay in human cells were determined using *t*-test. All in vitro experiments were repeated 3 times. A probability (*p*) value of 0.05 or less was considered to be the criterion for a significant difference. For correlation analysis, Person’s *r* method was used.

## Figures and Tables

**Table 1:** Effect of PEITC on cell viability after 6 and 24 h exposure.

PEITC (μM)	6 h		24 h	
	RAW264.7	SW480	RAW264.7	SW480
0	100 ± 0.066	100 ± 0.014	99.93 ± 0.067	100 ± 0.035
10	97.91 ± 0.072	101.14 ± 0.014	87.6 ± 1.25 **	78.26 ± 0.019 ***
20	98.59 ± 0.074	94.24 ± 0.035	75.17 ± 1.95 ***	78.04 ± 0.028 ***
40	98.97 ± 0.12	95.48 ± 0.023	57.7 ± 0.55 ***	64.44 ± 0.030 ***

Data are expressed as average percentage cell viability ± SEM (*n* ≥ 3). ** *p* < 0.01, *** *p* < 0.001 compared with positive-control (vehicle-DMSO treated cells not exposed to PEITC) cells. PEITC, phenethyl isothiocyanate; DMSO, dimethyl sulfoxide.

**Table 2:** Down-regulated inflammatory mediators in lipopolysaccharide induced macrophage and cancerous colon epithelial cells by 10 μM PEITC.

Gene Name Abbreviation	Full Gene Name	Partial GO Term (Geneontology.Org)	% Suppression in RAW264.7	% Suppression in SW480
*CCL2*	Chemokine (C–C motif) ligand 2	Inflammatory response; Chemokine activity	97.20	97.62
*CD40*	CD40 antigen	Signal transduction; Immune response; Apoptosis	91.77	54.73
*CXCL10*	Chemokine (C–X–C motif) ligand 10	Inflammatory response; Chemokine activity	96.67	93.01
*NFκB1*	Nuclear factor of kappa light chain gene enhancer in B-cells 1, p105	DNA binding; Regulation of transcription	95.35	43.69
*NFκBiα*	Nuclear factor of kappa light chain gene enhancer in B-cells inhibitor, alpha	Nucleus; Protein binding; Cytoplasm: Regulation of cell proliferation; Protein-nucleus import, translocation	87.82	38.44
*REL*	Reticuloendotheliosis oncogene	DNA binding; Regulation of transcription	82.64	28.80
*RELβ*	Avian reticuloendotheliosis viral (v-rel) oncogene related B	Transcription factor activity; Intracellular; T-helper 1 type immune response	73.54	66.80

**Table 3:** Primer sequences used in the study.

	RT-qPCR		ChIP-qPCR	
	RAW264.7	SW480		SW480
*CXCL10*	F: 5′-attctttaagggctggtctga-3′	F: 5′-gaaagcagttagcaaggaaaggt-3′		NA
	R: 5′-cacctccacatagcttacagt-3′	R: 5′-gacatatactccatgtagggaagtga-3′		
*CD40*	F:5′-acgagtcagactaatgtcatctgtg-3′	F: 5′-ggtctcacctcgctatggtt-3′		NA
	R:5′-ggtttcttgaccacctttttgat-3′	R: 5′-cagtgggtggttctggatg-3′		
*CCL2*	F: 5′-catccacgtgttggctca-3′	F: 5′-agtctctgccgcccttct-3′		NA
	R: 5′-gatcatcttgctggtgaatgagt-3′	R: 5′-gtgactggggcattgattg-3′		
*NFκB1*	F: 5′-gaggagaccggcaactca-3′	F: 5′-accctgaccttgcctatttg-3′		F:5′-ttggcaaaccccaaagag3′
	R: 5′-gtccatctccttggtctgct-3′	R: 5′-agctctttttcccgatctcc-3′		R:5′-ggtttcccacgatcgattt-3′
*βActin/GAPDH*	F: 5′-aaccgtgaaaagatgacccagat-3′	F: 5′-agccacatcgctcagacac-3′		NA
	R: 5′-cacagcctggatggctacgt-3′	R: 5′-gcccaatacgaccaaatcc-3′		

NA, not applicable.

## Abbreviations

AOM	Azoxymethane
CAC	Colitis Associated Cancer
CRC	Colorectal cancer
DSS	Dextran Sodium Sulfate
hDAI	histological Disease Activity Index
IBD	Inflammatory Bowel Disease
NFκB1	Nuclear factor kappa B1
PEITC	Phenethyl Isothiocyanate
UC	Ulcerative Colitis
vDAI	visible Disease Activity Index
